# A propensity score matching study on robot-assisted radical cystectomy for older patients: comparison of intracorporeal ileal conduit and cutaneous ureterostomy

**DOI:** 10.1186/s12894-022-01123-3

**Published:** 2022-11-07

**Authors:** Fumiya Kadoriku, Yutaro Sasaki, Kyotaro Fukuta, Yuichiro Atagi, Keito Shiozaki, Kei Daizumoto, Ryotaro Tomida, Yoshiteru Ueno, Megumi Tsuda, Yoshito Kusuhara, Tomoya Fukawa, Yutaka Yanagihara, Kunihisa Yamaguchi, Yasuyo Yamamoto, Hirofumi Izaki, Masayuki Takahashi, Sadamu Yamashi, Masaharu Kan, Hiroomi Kanayama

**Affiliations:** 1 Department of Urology, Tokushima University Graduate School of Biomedical Sciences, 3-18-15 Kuramoto-cho, 770-8503 Tokushima, Japan; 2 Department of Urology, Tokushima Prefectural Central Hospital, 1-10-3 Kuramoto-cho, 770-8539 Tokushima, Japan; 3 Department of Urology, Ehime Prefectural Central Hospital, 83 Kasuga-machi, 790-0024 Matsuyama, Japan

**Keywords:** Robot-assisted radical cystectomy, Intracorporeal ileal conduit, Older patients

## Abstract

**Background:**

Robot-assisted radical cystectomy (RARC) and intracorporeal urinary diversion are less invasive than conventional procedures. However, for older patients, cutaneous ureterostomy (CUS) may be preferred because urinary diversion using the intestine has a high incidence of perioperative complications and is highly invasive. The purpose of this study was to demonstrate the safety and efficacy of intracorporeal ileal conduit (ICIC) compared with CUS in patients aged 75 years or older who underwent RARC.

**Methods:**

From October 2014 to December 2021, 82 patients aged 75 years or older who underwent RARC at Tokushima University Hospital, Tokushima Prefectural Central Hospital, or Ehime Prefectural Central Hospital were retrospectively reviewed. Of these, 52 and 25 patients who underwent ICIC and CUS, respectively, were included. After adjusting the patients’ characteristics using propensity score-matching, surgical results and prognoses were retrospectively compared. The propensity score was based on age, Eastern Cooperative Oncology Group Performance Status Scale (ECOG-PS), American Society of Anesthesiologists physical status classification (ASA-PS), clinical tumor stage, and neoadjuvant chemotherapy.

**Results:**

The median age was lower in the ICIC group compared with the CUS group, and the proportion of high-risk cases (ECOG-PS ≥ 2 or ASA-PS ≥ 3) did not differ. The median operation time was longer in the ICIC group, and estimated blood loss was higher, compared with the CUS group. There were no significant differences in the incidence of complications within the first 30 postoperative days, incidence of complications 30–90 days after surgery, 2-year overall survival, 2-year cancer-specific survival, and 2-year recurrence-free survival. The stent-free rate was significantly lower in the CUS group than that in the ICIC group.

**Conclusion:**

In older patients, the ICIC group showed non-inferior surgical and oncological outcomes compared with the CUS group. Urinary diversion following RARC in older patients should be carefully selected by considering not only the age but also the general condition (including comorbidities) of the patient.

## Background

Robot-assisted radical cystectomy (RARC) with intracorporeal urinary diversion (ICUD) has the advantages of less bleeding, shorter hospital stay, and faster postoperative recovery compared with conventional procedures [1]. This minimally invasive surgery has become popular with many urologists. Nevertheless, for older patients, cutaneous ureterostomy (CUS) may be preferred because urinary diversion using the intestine has a high incidence of perioperative complications and is highly invasive. Therefore, to assess the safety and efficacy of RARC with intracorporeal ileal conduit (ICIC) in older patients, we investigated the perioperative and oncological outcomes of ICIC and CUS in patients aged 75 years or older who underwent RARC.

## Methods

From October 2014 to December 2021, 186 patients underwent RARC in Tokushima University Hospital, Tokushima Prefectural Central Hospital, and Ehime Prefectural Central Hospital. Of these, 82 patients aged 75 years or older were extracted. Five patients were excluded from this study because they underwent either extracorporeal ileal conduit (n = 2), intracorporeal orthotopic ileal neobladder (n = 2), or nephrostomy (n = 1). The final study cohort comprised 77 patients aged 75 years or older who underwent RARC with ICIC (n = 52 patients) or CUS (n = 25 patients). The Ethics Committee of each institution approved the study protocol, and all patients provided informed consent. To study the safety and efficacy of ICIC, we compared several perioperative variables between the ICIC and CUS groups, namely the patients’ characteristics, perioperative complications, and surgical and oncological outcomes. Complications were categorized in accordance with the Clavien–Dindo classification system. Grade ≥ 3 complications were defined as major complications, and grade ≤ 2 complications were defined as minor complications. Complications within 30 days after surgery were defined as early complications, and complications within 30–90 days after surgery were defined as late complications. Differences in patient characteristics between the two groups were adjusted by propensity score matching. The propensity score was based on potential confounders, namely age, Eastern Cooperative Oncology Group Performance Status Scale (ECOG PS), American Society of Anesthesiologists physical status classification (ASA-PS), clinical tumor stage, and neoadjuvant chemotherapy. The indications for performing CUS were the presence of comorbidities, history of radiotherapy to the abdomen, and patients’ preferences. Patients in both groups received the usual postoperative care, not the Enhanced Recovery After Surgery protocols. The single J stent was removed 2–4 weeks after surgery. The stent was reinserted in the event of urinary tract infection (UTI) or decreased renal function. In such cases, patients underwent stent replacement every 4 weeks.

### Statistical analysis

All continuous variables are expressed as median [interquartile range]. The t-test was used to analyze continuous variables, and Fisher’s exact test was used for nominal variables. Kaplan–Meier analysis and the log-rank test were also performed to compare the overall, cancer-specific, and recurrence-free survival of the two groups. A p value of < 0.05 was considered statistically significant. All statistical analyses were performed with EZR (Saitama Medical Center, Jichi Medical University, Saitama, Japan), which is a graphical user interface for R (ver.4.1.2, www.r-project.org).

## Results

**Fig. 1:**
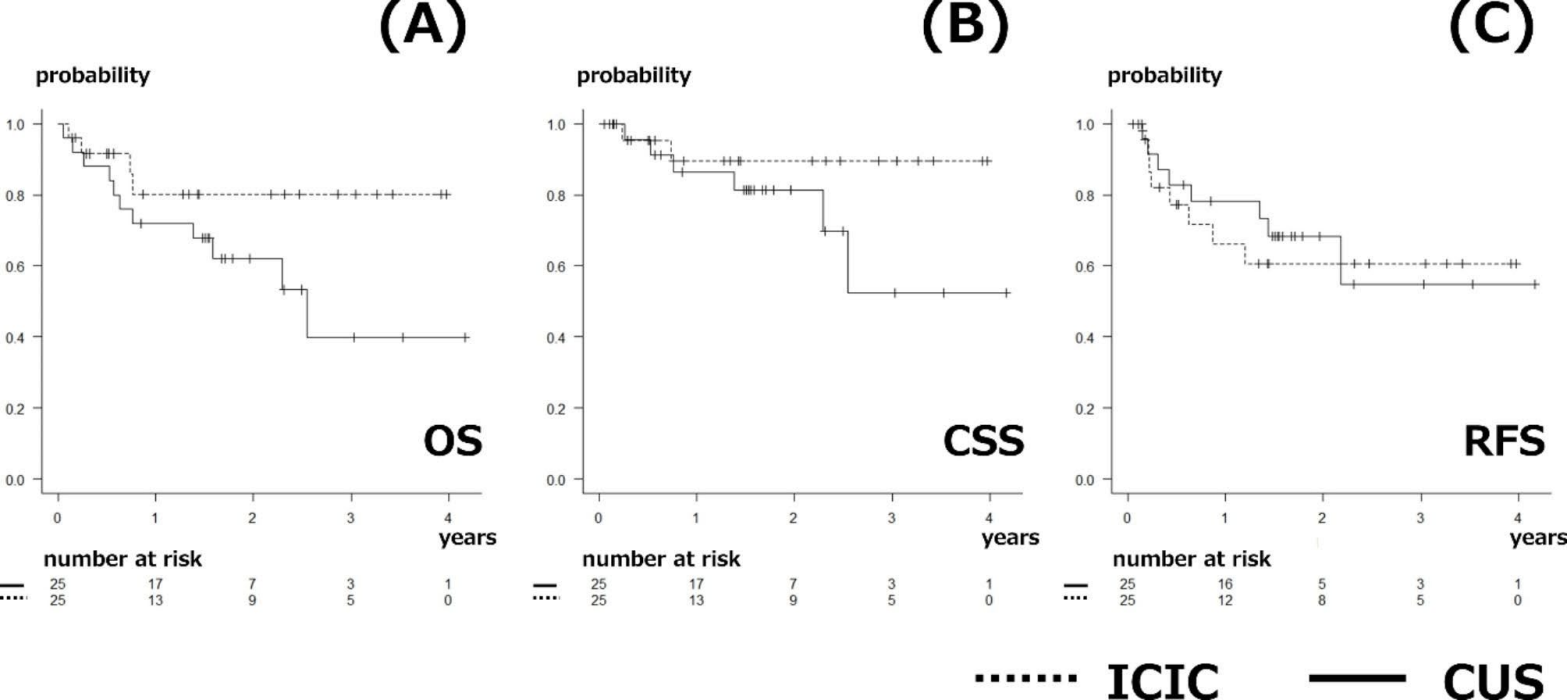
Kaplan–Meier curves of the intracorporeal ileal conduit (ICIC) and cutaneous ureterostomy (CUS) groups. The dashed line represents patients who underwent ICIC, and the solid line represents those who underwent CUS. (a) Overall survival (log-rank, p = 0.107), (b) cancer-specific survival (log-rank, p = 0.193), and (c) recurrence-free survival (log-rank, p = 0.762)

Table 1 shows the clinical characteristics of the patients. In the unmatched cohort, patients in the CUS group were significantly older than those in the ICIC group (median [interquartile range]: 83 [80–85] vs. 79 [76–81] years, respectively, p < 0.001), had a significantly lower proportion of clinical stage 2 or deeper tumors (68% vs. 92%, respectively, p = 0.015), and had a significantly lower preoperative chemotherapy rate (28% vs. 77%, respectively, p < 0.001). In the matched cohort after propensity score matching, the significant differences between the CUS and ICIC groups disappeared, except for age (83 [80–85] vs. 81 [76–83] years, respectively, p = 0.014). In the CUS group, patients underwent bilateral (n = 13), double-barrel (n = 7), and single (n = 5) ureterostomy. Table 2 shows the surgical outcomes in both groups after propensity score matching. Although the radical cystectomy time did not significantly differ between the two groups, the overall operative time was significantly longer in the ICIC group vs. the CUS group (463 [440–530] vs. 288 [256–360] min, respectively, p < 0.001). The estimated blood loss (EBL) was significantly higher in the ICIC vs. CUS group (392 [194–537] vs. 150 [106–275] ml, respectively, p = 0.011). In addition, the time to first liquids (2 [1–3] vs. 1 [1] postoperative days, p < 0.001) and time to first meal (4 [3–5] vs. 2 [2] postoperative days, p < 0.001) were significantly longer in the ICIC group vs. the CUS group, respectively. Although the time to flatus was significantly longer in the ICIC group vs. the CUS group (2 [2, 3] vs. 1 [1, 2] postoperative days, respectively, p = 0.001), there was no difference in the time to first bowel movement between the groups (3 [3–5] vs. 4 [3–5] postoperative days, respectively, p = 0.437). There were no significant differences in the incidences of early or late complications between the two groups. The incidences of gastrointestinal complications, namely ileus, ileo-ureteric anastomotic leak, and conduit–enteric fistula, were significantly higher in the ICIC group vs. the CUS group, as early complications (28% vs. 4%, respectively, p = 0.049). No gastrointestinal complications were observed as late complications. The incidence of UTI as an early complication did not differ between the ICIC and CUS groups (4% vs. 16%, respectively, p = 0.349). The incidence of UTI as a late complication tended to be higher in the CUS group vs. the ICIC group (20% vs. 0%, respectively, p = 0.050). There were no significant differences in early or late readmission and reoperation rates between the groups. However, the stent-free rate was significantly lower in the CUS group vs. the ICIC group (12% vs. 100%, respectively, p < 0.001). Figure 1 shows the overall, cancer-specific, and recurrence-free survival rates. The 2-year survival rates did not differ between the ICIC and CUS groups: overall survival (80% vs. 62%, respectively, p = 0.107), cancer-specific survival (90% vs. 81%, respectively, p = 0.193), and recurrence-free survival (61% vs. 68%, respectively, p = 0.762).

**Table 1:** Clinical characteristics of patients in the ICIC and CUS groups in unmatched and matched cohorts

Clinical characteristics											
	Unmatched Cohort				p value		Matched Cohort				p value
	ICIC		CUS				ICIC		CUS		
Number of patients	52		25				25		25		
Age, years, median (IQR)	79 (76-81)		83 (80-85)		<0.001		81 (76-83)		83 (80-85)		0.014
Male, n (%)	40	(76.9)	17	(68.0)	0.418		18	(72.0)	17	(68.0)	1.000
BMI, kg/m^2^, median (IQR)	23.5 (21.0-25.4)		24.3 (21.4-25.7)		0.440		23.3 (20.9-25.3)		24.3 (21.4-25.7)		0.410
ECOG-PS ≥2, n (%)	2	(3.8)	4	(16.0)	0.083		1	(4.0)	4	(16.0)	0.349
ASA-PS ≥3, n (%)	14	(26.9)	10	(40.0)	0.297		8	(32.0)	10	(40.0)	0.769
Prior abdominal surgery, n (%)	22	(42.3)	8	(32.0)	0.459		11	(44.0)	8	(32.0)	0.561
Clinical tumor stage <2, n (%)	4	(7.7)	8	(32.0)	0.015		3	(12.0)	8	(32.0)	0.171
Clinical tumor stage ≥2, n (%)	48	(92.3)	17	(68.0)			22	(88.0)	17	(68.0)	
Neoadjuvant chemotherapy, n (%)	40	(76.9)	7	(28.0)	<0.001		14	(56.0)	7	(28.0)	0.085

ICIC: intracorporeal ileal conduit; CUS: cutaneous ureterostomy

IQR: interquartile range

BMI: body mass index

ECOG-PS: Eastern Cooperative Oncology Group performance status

ASA-PS: American Society of Anesthesiologists physical status classification

**Table 2:** Surgical outcomes of patients in the ICIC and CUS groups in the matched cohort

Surgical outcomes						
	ICIC		CUS		p value	
	(n = 25)		(n = 25)			
Overall operative time, min, median (IQR)	463 (440–530)		288 (256–360)		<0.001	
Radical cystectomy time, min, median (IQR)	180 (146–200)		164 (136–205)		0.731	
Estimated blood loss, ml, median (IQR)	392 (194–537)		150 (106–275)		0.011	
Transfusion, n (%)	9	(36.0)	4	(16.0)	0.196	
Pathological tumor stage < 2, n (%)	10	(40.0)	9	(36.0)	1.000	
Pathological tumor stage ≥ 2, n (%)	15	(60.0)	16	(64.0)		
Lymph node yield, median (IQR)	15 (8–21)		13 (7–18)		0.426	
Positive lymph node, n (%)	7	(28.0)	3	(12.0)	0.289	
Time to liquid, POD, median (IQR)	2 (1–3)		1 (1–1)		<0.001	
Time to meal, POD, median (IQR)	4 (3–5)		2 (2–2)		<0.001	
Time to flatus, POD, median (IQR)	2 (2–3)		1 (1–2)		0.001	
Time to bowel, POD, median (IQR)	3 (3–5)		4 (3–5)		0.437	
Hospital stay, days, median (IQR)	26 (20–35)		21 (16–29)		0.093	
30-d Complication	12	(48.0)	10	(40.0)	0.776	
Minor complication, n (%)	6	(24.0)	5	(20.0)	1.000	
Major complication, n (%)	6	(24.0)	5	(20.0)	1.000	
30-d Readmission, n (%)	1	(4.0)	1	(4.0)	1.000	
30-d Reoperation, n (%)	1	(4.0)	0	(0.0)	1.000	
90-d Complication	4	(16.0)	7	(28.0)	0.496	
Minor complication, n (%)	3	(12.0)	4	(16.0)	1.000	
Major complication, n (%)	1	(4.0)	3	(12.0)	0.609	
90-d Readmission, n (%)	2	(8.0)	6	(24.0)	0.247	
90-d Reoperation, n (%)	1	(4.0)	0	(0.0)	1.000	
Stent free, n (%)	25	(100.0)	3	(12.0)	<0.001	

ICIC: intracorporeal ileal conduit; CUS: cutaneous ureterostomy

IQR: interquartile range

POD: postoperative day

## Discussion

RARC using the da Vinci Surgical System™ (Intuitive Surgical, Sunny vale, CA, USA) was first reported in 2003 by Menon et al. [2]. Owing to the high flexibility and sophisticated operability of the instruments, this system is effective in radical cystectomy, which requires high surgical skill. In Japan, RARC has spread rapidly since it was covered by health insurance in April 2018.

There are several reports of RARC in older patients[3, 4]. Phillips et al. reported perioperative outcomes in 22 cases of RARC in older patients aged 80 years or older [3]. With a 90-day complication rate of 34.8% and no cases of Clavien–Dindo grade 5 complications, the authors concluded that RARC should be strongly considered in patients aged 80 years or older who are candidates for cystectomy. De Groote et al. reported on the perioperative outcomes and prognoses of 22 older patients aged 80 years or older among 155 patients who underwent RARC [4]. The older and younger patients did not differ in the rates for perioperative complications, mortality, or 3-year recurrence-free survival. Therefore, the authors concluded that skilled operators can safely perform RARC in older patients. Elsayed et al. reported on the perioperative and oncological outcomes of 81 older adult patients aged 80 years or older among 522 patients who underwent RARC [5]. Despite higher Charlson Comorbidity Index and ASA-PS scores in the older patients compared with younger patients, there was no significant difference in terms of blood loss, overall and major complications, readmissions, or perioperative mortality. Older patients exhibited comparable 5-year recurrence-free survival and cancer-specific survival compared with younger patients. The authors concluded that RARC did not increase perioperative risks or compromise oncological outcomes in older patients and should be considered a treatment option in this population.

The choice of urinary diversion following radical cystectomy in older patients is controversial. Ileal conduit is usually avoided in older and more frail patients because of its longer operative time and hospital stay and higher blood loss, transfusion rates, necessity of intensive care, and incidence of perioperative complications (including gastrointestinal complications), compared with CUS [6]. Conversely, ICUD following RARC has the advantages of less bleeding, shorter hospital stay, and faster postoperative recovery, compared with conventional procedures [1]. However, there are few reports of ICIC in older patients, and thus the safety of ICIC in older patients has not been clarified.

Compared with CUS, ICIC involves many more complicated surgical procedures and consequently a longer operation time. In the current study, ICIC was associated with a significantly prolonged operation time and increased EBL compared with CUS. However, the transfusion rate did not differ significantly from that of the CUS group. Mastroianni et al. reported the results of the randomized controlled trial comparing 58 cases of open radical cystectomy (ORC) and 58 cases of RARC with ICUD [7]. Both EBL (401 [243–511] vs. 467 [330–625] ml, p = 0.020) and the transfusion rate (22% vs. 41%, p = 0.046) were significantly lower in the RARC group vs. the ORC group, respectively. In the current study, the EBL in the ICIC group was 392 [194–537] ml, with a transfusion rate of 36%. Compared with the results of the study by Mastroianni et al., the transfusion rate in our study was higher despite similar blood loss in both studies. In the study by Mastroianni et al., the age of the patients who underwent RARC was 64 [53–70] years, which is much younger compared with the patients in our study, and younger patients may have a higher tolerance to hemorrhage. In addition, there were no significant differences in the length of hospital stay or the incidence of perioperative complications in the current study, suggesting that the effect of prolonging the operation time was tolerable. Conversely, there were no significant differences between the two groups in the overall, cancer-specific, or recurrence-free survival rates. Based on these results, we believe that ICIC is comparable to CUS in terms of oncological prognoses and perioperative outcomes, and that ICIC can be performed safely even in older patients.

No reports have evaluated the health-related quality of life (HRQoL) of patients between ICIC and CUS, although several reports have evaluated the HRQoL of patients who underwent radical cystectomy [8, 9]. Mastroianni et al. reported a comparison of patient-reported HRQoL scores between ORC and RARC with ICUD [8]. Overall, both groups reported significant worsening of body image and physical and sexual functions at the 1-year follow-up compared with baseline. Comparing the two groups, patients who underwent ORC were more likely to experience a decline in role functioning and report higher scores on the symptoms scale, while those who underwent RARC with ICUD were more likely to report significant increases in urinary symptoms and related problems. Arman et al. reported a comparison of postoperative HRQoL after ileal conduit and CUS after ORC in 70 patients [9]. The authors concluded that the HRQoL after ileal conduit was significantly superior to that after bilateral but not unilateral CUS. The authors also reported a stent-free rate of 73.9% and 32.0% for bilateral and unilateral CUS, respectively [9]. In the current study, the stoma was created by the Toyoda method in the CUS group [10]. Nevertheless, the stent-free rate was significantly lower in the CUS group compared with the ICIC group. In CUS cases that do not become stent-free, the burden on patients and their families forced to undergo regular stent replacement cannot be underestimated. Improving the stent-free rate is an urgent need from the perspective of UTIs. Murai et al. reported the effects of indwelling stents on urinary bacterial flora and UTI in 24 patients who underwent ureterostomy [11]. Patients with indwelling stents had higher incidences of UTI and recurrent UTI compared with patients who were stent-free. In addition, patients with UTIs often have stent obstruction, which increases intrapelvic pressure and causes UTI. Murai et al. concluded that indwelling stents after CUS are strong risk factors for pyelonephritis and the development of antibiotic-resistant bacteria. In the current study, the incidence of UTI tended to be higher in the CUS group compared with the ICIC group within 30–90 days after surgery. Thus, the low incidence of UTI is also a significant aspect of ICIC. Based on the above, the lower stent-free rate and high UTI rate in the CUS group may lead to decreased postoperative HRQoL of patients. However, we did not compare HRQoL between the ICIC and CUS groups in this study. A future research topic is to conduct an objective evaluation of HRQoL using questionnaires. Considering the higher stent-free rate and lower UTI rate (also in the older patients), ileal conduit is preferable to CUS. However, there are patients who cannot undergo ileal conduit for reasons such as their general condition and comorbidities. In such cases, further efforts are required to improve the stent-free rate. For example, several reports have evaluated tubeless CUS during open surgery [12–15]. However, we did not find any reports on CUS in robot-assisted surgery. Whether the tubeless surgical technique used during open surgery can be applied to robot-assisted surgery is a topic for future studies.

The present study had some limitations. First, this was a retrospective study with a small sample size. Second, although the patient backgrounds were matched as much as possible by propensity score matching, it was not possible to match the patients’ ages, and patient selection bias was unavoidable. Third, we did not objectively evaluate the patients’ HRQoL between the ICIC and CUS groups. Finally, the assessment of the general condition of older patients was inadequate. ECOG-PS and ASA-PS are simple evaluation tools for comprehensively evaluating a patient’s general condition. However, in older patients, factors such as decreased physical reserve, decreased cognitive function, and comorbidities may make it difficult to assess the general condition using these assessment tools alone. In the future, it may be more practical to use screening tools such as the G8 and the Flemish version of the Triage Risk Screening Tool to evaluate the general condition of older patients [16].

## Conclusion

In older patients in this study, the ICIC group showed non-inferior surgical and oncological outcomes compared with the CUS group. Urinary diversion following RARC in older patients should be carefully selected by considering not only the age but also the general condition (including comorbidities) of the patient.

## Data Availability

The datasets used and/or analyzed during the current study are available from the corresponding author on reasonable request.
